# Comparison of Clinical Outcomes of Radical Prostatectomy versus IMRT with Long-Term Hormone Therapy for Relatively Young Patients with High- to Very High-Risk Localized Prostate Cancer

**DOI:** 10.3390/cancers13235986

**Published:** 2021-11-28

**Authors:** Hung-Jen Shih, Shyh-Chyi Chang, Chia-Hao Hsu, Yi-Chu Lin, Chu-Hsuan Hung, Szu-Yuan Wu

**Affiliations:** 1 Division of Urology, Department of Surgery, Changhua Christian Hospital, Changhua 500, Taiwan; 106385@w.tmu.edu.tw; 2 Department of Recreation and Holistic Wellness, MingDao University, Changhua 500, Taiwan; 3 Department of Urology, School of Medicine, College of Medicine, Taipei Medical University, Taipei 110, Taiwan; 4 Department of Urology, Lo-Hsu Medical Foundation, Lotung Poh-Ai Hospital, Yilan 265, Taiwan; mork2747@gmail.com (S.-C.C.); c095001@mail.pohai.org.tw (C.-H.H.); ret7988@mail.pohai.org.tw (Y.-C.L.); c857026@mail.pohai.org.tw (C.-H.H.); 5 Faculty of Medicine, National Yang-Ming University School of Medicine, Taipei 11221, Taiwan; 6 Department of Food Nutrition and Health Biotechnology, College of Medical and Health Science, Asia University, Taichung 413, Taiwan; 7 Big Data Center, Lo-Hsu Medical Foundation, Lotung Poh-Ai Hospital, Yilan 265, Taiwan; 8 Division of Radiation Oncology, Lo-Hsu Medical Foundation, Lotung Poh-Ai Hospital, Yilan 265, Taiwan; 9 Department of Healthcare Administration, College of Medical and Health Science, Asia University, Taichung 413, Taiwan; 10 Cancer Center, Lo-Hsu Medical Foundation, Lotung Poh-Ai Hospital, Yilan 265, Taiwan; 11 Graduate Institute of Business Administration, College of Management, Fu Jen Catholic University, Taipei 242062, Taiwan; 12 Department of Management, College of Management, Fo Guang University, Yilan 262307, Taiwan; 13 Centers for Regional Anesthesia and Pain Medicine, Wan Fang Hospital, Taipei Medical University, Taipei 110, Taiwan

**Keywords:** prostate cancer, radical prostatectomy, intensity-modulated radiotherapy, young men

## Abstract

**Simple Summary:**

That the definitive optimal treatments for relatively young men (aged ≤ 65 years) with high- or very high-risk localized prostate cancer (HR/VHR-LPC) are radical prostatectomy (RP) or radiation plus antiandrogen therapy (RT-ADT) is controversial. To the best of our knowledge, our study is the first and largest to examine biochemical failure (BF), all-cause death, locoregional recurrence, and distant metastasis in relatively young men with HR/VHR-LPC as defined by National Comprehensive Cancer Network risk strata. After head-to-head propensity score matching was used to balance the potential confounders, a multivariable Cox proportional hazards regression model was used to analyze oncologic outcomes. In relatively young men with HR/VHR-LPC, RP and RT-ADT yielded similar oncologic outcomes and RP reduced the risk of BF compared with RT-ADT.

**Abstract:**

That intensity-modulated radiotherapy (IMRT) plus antiandrogen therapy (IMRT-ADT) and radical prostatectomy (RP) are the definitive optimal treatments for relatively young patients (aged ≤ 65 years) with high- or very high-risk localized prostate cancer (HR/VHR-LPC), but remains controversial. We conducted a national population-based cohort study by using propensity score matching (PSM) to evaluate the clinical outcomes of RP and IMRT-ADT in relatively young patients with HR/VHR-LPC. *Methods*: We used the Taiwan Cancer Registry database to evaluate clinical outcomes in relatively young (aged ≤ 65 years) patients with HR/VHR-LPC, as defined by the National Comprehensive Cancer Network risk strata. The patients had received RP or IMRT-ADT (high-dose, ≥72 Gy plus long-term, 1.5–3 years, ADT). Head-to-head PSM was used to balance potential confounders. A Cox proportional hazards regression model was used to analyze oncologic outcomes. *Results*: High-dose IMRT-ADT had a higher risk of biochemical failure (adjusted hazard ratio [aHR] = 2.03, 95% confidence interval [CI] 1.56–2.65, *p* < 0.0001) compared with RP; IMRT-ADT did not have an increased risk of all-cause death (aHR = 1.2, 95% CI 0.65–2.24, *p* = 0.564), locoregional recurrence (aHR = 0.88, 95% CI 0.67–1.06, *p* = 0.3524), or distant metastasis (aHR = 1.03, 95% CI 0.56–1.9, *p* = 0.9176) compared with RP. *Conclusion*: In relatively young patients with HR/VHR-LPC, RP and IMRT-ADT yielded similar oncologic outcomes and RP reduced the risk of biochemical failure compared with IMRT-ADT.

## 1. Introduction

According to estimates from Global Cancer Statistics 2020, prostate cancer (PC) is the second most common cancer (1,414,259 new cases) and the fifth leading cause of cancer-related deaths (375,304 deaths) in men worldwide [1]. According to the Taiwan Cancer Registry database (TCRD), PC is the fifth most common cancer and the sixth leading cause of cancer-related deaths in men in Taiwan [2,3]. Increased treatment efficacy and decreased treatment-related side effects are the most critical concerns for patients with PC [4], especially younger patients whose survival is expected to be relatively long. For localized PC (LPC), therapeutic treatment decision-making is based on the LPC risk stratification and health status of the patient [5,6,7]. Several risk stratification systems, such as those of D’Amico, the American Urological Association (AUA), the European Association of Urology (EAU), and the National Comprehensive Cancer Network (NCCN) [8] include various factors such as prostate-specific antigen (PSA), biopsy Gleason score, or clinical T stage, which are used to classify patients and provide crucial data for treatment modality decision-making [5,6,7]. The NCCN risk stratification system [8] is used by most physicians in Taiwan. Patients with high-risk or very high-risk LPC (HR/VHR-LPC) have poor oncologic outcomes compared with very low-, low-, favorable-intermediate-, or unfavorable-intermediate-risk LPC, and consequently, these patients require definitive therapy such as external beam radiotherapy (EBRT) or radical prostatectomy (RP) rather than watchful waiting unless the expected survival of the patient is less than five years and they are asymptomatic [8,9,10]. Recently, the proportional rate of high-risk LPC (HRLPC) has increased (from 11.8% in 2004 to 20.4% in 2016) [11]. However, no consensus has been reached yet on the optimal treatment recommendation for HR/VHR-LPC to be included in clinical guidelines [5,7].

According to the European Association of Urology–European Society for Radiotherapy and Oncology–International Society of Geriatric Oncology 2020 guidelines on PC, a reasonable first-step treatment for patients with HRLPC includes RP or dose-escalated intensity-modulated radiotherapy (IMRT) plus long-term (2–3 years) androgen-deprivation therapy (ADT) [7]. The NCCN version 2.2021 guidelines [8] indicate that the treatment of choice for patients with HR/VHPC with a life expectancy ˃ 5 years is EBRT plus 1.5–3 years of ADT, EBRT plus brachytherapy, including 1–3 years of ADT or RP [8]. However, no randomized clinical trials (RCTs) have yet to evaluate high-dose IMRT plus ADT versus RP regarding oncologic outcomes. Current recommendations for deciding on a treatment modality are based on the results of retrospective population-based studies or meta-analyses [8,9,10]. However, the comparisons in these studies [8,9,10] of the oncologic outcomes of RP and high-dose IMRT plus ADT for patients with HR/VHR-LPC are subject to some concerns, with their analyses of relatively young patients with expected long-term survival being particularly questionable. The most common concern is selection bias among patients who are receiving RP and RT because of differing backgrounds, inconsistent irradiation doses, varying durations of ADT use, and distinct RT techniques [8,9,10]. For retrospective population studies, an effort should be made to balance baseline patient characteristics and maintain consistency in risk classifications and treatment protocols.

For relatively young patients with HRLPC who received definitive treatment, more favorable oncologic outcomes were noted in patients who received RP than in those who received RT [12,13]. However, the results of these studies should be cautiously interpreted because the proportion of ADT used was not recorded in these studies [12,13], and patients who received RP were younger and had fewer comorbidities and less advanced tumors compared with patients who received RT in retrospective studies [14,15]. Furthermore, detailed comparative studies of all-cause death, locoregional recurrence (LRR), biochemical failure (BF), and distant metastasis (DM) associated with standard treatments (RP versus RT plus long-term ADT) in relatively young patients with HRLPC are still lacking. High-dose IMRT plus long-term ADT or RP is one of the treatment recommendations for patients with NCCN HR/VHR-LPC according to the NCCN guidelines [8]. Administering the treatment that can provide the optimal survival benefit is the paramount concern when treating relatively healthy younger (aged ≤ 65 years) patients with PC. Patients with PC aged < 65 years were defined as young in our study because the mean age of patients diagnosed as having PC is 69 years in Taiwan [3], and the mean age of patients diagnosed with PC is 66 years in the United States [16]. Several studies have compared RP and EBRT with or without ADT in relatively young and healthy patients, and the results have demonstrated that RP provides superior survival outcomes compared with EBRT [12,13,17]. However, varying definitions of risk stratification, various ADT duration, and inconsistent radiation dosages are concerns that militate against applying the results of these studies in current clinical suggestions [12,13,17]. Therefore, a head-to-head propensity score matching (PSM) analysis was conducted in this study to evaluate these oncologic outcomes of RP and compare them with high-dose IMRT plus long-term ADT for patients with HR/VHR-LPC according to the NCCN risk stratification system [8].

## 2. Patients and Methods

### 2.1. Database

A population-based cohort study using Taiwan’s National Health Insurance Research Database (NHIRD), which is linked to the TCRD, was conducted. The TCRD contains data of nearly 100% of cancer patients in Taiwan, which was established in 1979 [18]. The NHIRD includes de-identified basic demographic information, disease diagnoses, drugs, and procedures of all beneficiaries [19]. To verify the cause of death and vital status of each patient, The TCRD death registry was additionally linked to the NHIRD. The detailed data on LPC, such as the American Joint Committee on Cancer (AJCC) stage, surgical procedures, techniques, RT dose, hormone treatments, and pathologic stages, were included in TCRD [2,20,21,22,23].

### 2.2. The Cohort

We enrolled patients identified from the TCRD to establish a cohort. Relatively young patients (aged ≤ 65 years) who had received a diagnosis of NCCN HR/VHR-LPC and received high-dose IMRT and long-term (1.5–3 years) ADT or RP between 1 January 2011 and 31 December 2016 were included. In this cohort, the relatively young men with HR/VHR-LPC and a life expectancy > years received combination IMRT and long-term ADT or RP in accordance with NCCN guidelines [8]. The index date was defined as the date of LPC diagnosis by pathological confirmation. The patients were followed from the index date to 31 December 2018. Our protocols were reviewed and approved by the Institutional Review Board (IRB109-015-B). The specific method is stated in our previous paper [2].

#### 2.2.1. Inclusion Criteria

(1) Tumor staging from cT1 to T3a, pretreatment PSA levels from 0 to more than 20 ng/mL, or grade group from 1 to 5 were defined as NCCN HR/VHR-LPC.
(2) A newly diagnosed NCCN HR/VHR-LPC who received RP or IMRT.
(3) No other cancer, clinical lymph node metastasis, or distant metastasis were named as LPC.
(4) Removal of the entire prostate gland, seminal vesicles, and the surrounding lymph nodes was defined as standard surgical procedures of RP [24].
(5) Standard IMRT was defined that pelvic lymph nodes receiving prophylactic doses of 45 Gy in 1.8 Gy per fraction, the seminal vesicles having 54 Gy, and the prostate receiving boost radiation dose to 72–81 Gy.

#### 2.2.2. Exclusion Criteria

(1) Doses less than 72 Gy of IMRT were defined as insufficient irradiation doses based on previous reports and NCCN guidelines [8,25,26,27].
(2) IMRT without long-term (<1.5 years) ADT.
(3) Patients with PC who did not receive standard RP or doses of IMRT after LPC diagnosis.

BF after RP was defined as a serum PSA level of ≥0.2 ng/mL according to the definition of BF of the AUA [28]. BF was defined as a PSA nadir plus ≥2 ng/mL after having reached a PSA nadir after treatment of IMRT based on the Radiation Therapy Oncology Group-ASTRO Phoenix Consensus [29]. However, the possible treatments (such as salvage irradiation after RP, salvage prostatectomy, high-intensity focused ultrasound after IMRT, or systemic therapy after BF) were allowed and did not disqualify patients from our inclusion. The clinical outcomes (BF, LRR, DM, and all-cause death) were compared between patients who received RP (group 1) and high-dose IMRT-ADT (group 2). The LRR or DM was defined clinically or radiologically as overt local recurrence or distant failure. Local recurrence was confirmed by prostate biopsy through pathological diagnosis.

### 2.3. Covariates

The covariates, which might be associated with all-cause death, are shown in Table 1. Comorbidities were scored by the Charlson comorbidity index (CCI) scores [30,31] and special comorbidities associated with all-cause death. Comorbidities censored 12 months before the index date were included in our study. If the primary diagnostic code, using the *International Classification of Diseases, Ninth Revision, Clinical Modification* (*ICD-9-CM*), upon visit to the first admission or the outpatient department, was repeated more than twice, comorbidities were included and verified in our study. We removed peripheral vascular disease, cerebrovascular disease, chronic pulmonary disease, myocardial infarction, congestive heart failure, diabetes, and hypertension from CCI scores to prevent repeated adjustment.

### 2.4. Endpoints

All-cause death between RP and high-dose IMRT plus long-term ADT is our primary endpoint. BF, LRR, and DM between young men with HR/VHR-LPC who underwent RP and those who underwent high-dose IMRT plus long-term ADT were our secondary endpoints.

### 2.5. Propensity Score Matching

We used optimal matching in our study as a 1:4 ratio to reach a sufficient sample size for further analysis [32]. If the sample size was insufficient for a 1:4 ratio, we used a 1:3 ratio to increase the sample size for analysis. Nevertheless, not all covariates were 1:3 matched between the RP and IMRT groups; some covariates were matched 1:2 or 1:1 between the RP and IMRT groups. Thus, an exact 3:1 ratio between the RP and IMRT groups was not attained.

### 2.6. Statistics

In modeling the study duration from the index date to all-cause mortality, a cox proportional hazards model was applied with the control for confounders in young patients with NCCN HR/VHR-LPC. To minimize the influences of potential confounders, head-to-head PSM was conducted during comparisons of treatment outputs between those two treatment groups. A width equal to 0.2 of the standard deviation of the logit of the propensity score was balanced in the logit of the propensity score using calipers [33,34]. In order to reduce any discrepancy between the two treatment groups, the controls with similar background covariates to the case patients were opted for [35]. A strong and robust predictor was applied to account for clustering within matched sets, and a Cox model was applied to regress endpoints on the treatment status. Thereafter, the multivariable Cox regression analysis was used to calculate the hazard ratios (HRs) to define whether the covariates were required to be re-adjusted to diminish any confounding effects if there was an unbalance in conditions existing after PSM was performed. Potential prognosis factors were also tightly controlled during the analysis, and the endpoint was all factors associated with the mortality in the treatment group.

The risk of all-cause death was calculated for young men with HR/VHR-LPC. The other secondary endpoints, such as BF, LRR, and DM, were assessed and estimated by applying a proportional subdistribution hazard regression model to cope with the competing risk of death in the analysis of time-to-event data. All statistical analyses were conducted using SAS version 9.3. *p* < 0.05 was considered as significant in a two-tailed Wald test. The risk of all-cause death was also estimated by applying the Kaplan-Meier method, and differences among high-dose IMRT + HT or RP were defined using the stratified log-rank test to compare survival curves (stratified on matched sets). A *p*-value less than 0.05 was considered statistically significant.

## 3. Results

### 3.1. Study Cohort after Propensity Scores Matching

We included 696 young men with NCCN HR/VHR-LPC (Table 1), 481 receiving RP, and 215 receiving high-dose IMRT-ADT groups, respectively. The mean follow-up duration for the RP and IMRT + long-term HT groups were 60.2 and 59.9 months after the index dates, successively. After PSM was performed, there were no statistically significant differences (*p* > 0.05) noticed between groups of covariates (Table 1). Most *p*-values were more than 0.5, suggesting that the matching variables’ distribution was close (Table 1).

### 3.2. Clinical Outcomes between the Two Therapeutic Groups 

Treatment was not a significant predictor of all-cause mortality based on the multivariate Cox regression analysis (Table 2). RP was not associated with higher overall survival (OS) compared with the definitive high-dose IMRT-ADT in young patients with NCCN HR/VHR-LPC through multivariate Cox regression analysis. No significant differences were found in the descriptive covariates, except for hospital level and EAU risk group, because PSM was conducted accurately (Table 2). The adjusted hazard ratio (aHR; 95% confidence interval [CI]) of BF for IMRT-ADT compared with RP was 2.03 (1.56–2.65, *p* < 0.0001; Table 3). In younger patients with NCCN HR/VHR-LPC, RP did not significantly affect LRR compared with IMRT-ADT (Table 4). There were no significant differences for DM between IMRT-ADT and RP in younger patients with NCCN HR/VHR-LPC (Table 5). Taken together, IMRT-ADT was not a significant risk factor of all-cause death (aHR = 1.2, 95% CI 0.65–2.24, *p* = 0.564), LR (aHR = 0.88, 95% CI 0.67–1.06, *p* = 0.3524), or DM (aHR = 1.03, 95% CI 0.56–1.9, *p* = 0.9176) compared with RP. Hospital level and EAU risk group were significant prognostic factors for mortality, BF, and LRR by multivariate analysis (Table 2, Table 3 and Table 4). Moreover, through multivariate analysis, the EAU risk group was also a significant prognostic factor for DM (Table 5). 

**Figure 1:**
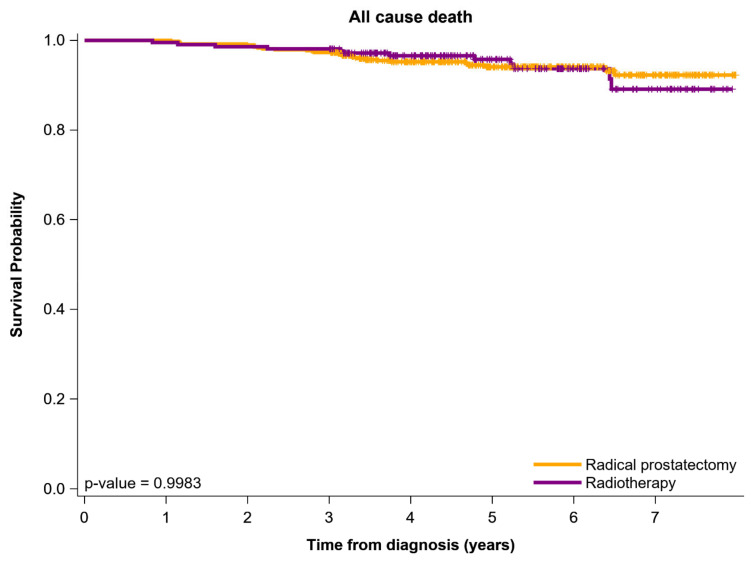
Survival curves for endpoints by Kaplan–Meier method for propensity score-matched young patients with NCCN high- to very high-risk prostate adenocarcinoma receiving various curative-intent treatments NCCN, National Comprehensive Cancer Network.

Kaplan–Meier survival curves for the PSM cohort of younger patients with NCCN HR/VHR-LPC who received high-dose IMRT-ADT or RP are presented in Figure 1. The survival curve for RP was not significantly better than that for high-dose IMRT-ADT in younger patients with NCCN HR/VHR-LPC. The 5-year survival rates for RP and high-dose IMRT-ADT were 94.7% and 95.9% (*p* = 0.9983), respectively.

## 4. Discussion

A well-designed study to compare the oncological outcomes between RP and high-dose IMRT plus long-term ADT in relatively young (≤ 65 years) men with NCCN HR/VHR-LPC based on the commonly used NCCN risk classifications remains lacking. Our study included patients with HR/VHR-LPC diagnosed according to the definition in the NCCN version 2.2021 guidelines [8] who received adequate long-term ADT and a sufficient radiation dosage in this well-designed PSM study. The findings of our study revealed that either RP or high-dose IMRT plus long-term ADT yield the same rate of all-cause death, LRR, and DM for relatively young men with NCCN HR/VHR-LPC after a mean 5-year follow-up duration. However, BF-free survival (BFFS) in the young men with NCCN HR/VHR-LPC who were receiving RP was superior to that of those patients receiving high-dose IMRT plus long-term ADT.

BF is a crucial endpoint when evaluating the efficacy of primary treatment, and patients with BF should receive salvage treatment to prevent or delay disease progression [36]. Patients with BF have an increased likelihood of experiencing substantial anxiety and negative moods and a decreased quality of life after salvage treatment [37,38]. Although the impact of BF on subsequent PC mortality remains unknown, an observational study found that BF was associated with an increased risk of PC mortality [39]. For relatively young healthy patients, therapy with low BF is a superior choice because of those patients’ relatively long life expectancy, according to the results of our study (Table 3). Relatively young patients with HR/VHR-LPC who were treated with high-dose IMRT plus long-term ADT experienced a 2.03-fold BF increase compared with those treated with RP. Although the effect of local treatment on BF was not observed in all-cause death, LRR, or DM after PSM (Table 2, Table 3, Table 4 and Table 5), this may be attributable to salvage treatment improving survival outcomes after BF of primary treatment and a long natural disease history to mortality of LPC [40,41,42,43,44]. Our study is compatible with the previous studies, which demonstrated no significant differences in all-cause death between RP or RT-ADT in men with HR/VHR-LPC [45,46]. Initial treatment with RP as compared with EBRT and ADT was not associated with an increased risk of prostate cancer-specific mortality in men with a Gleason score of 8-10 for prostate cancer [45]. Evidence demonstrating definitive superiority of either modality is lacking [46]. In our novel findings, in relatively young men with HR/VHR-LPC and a life expectancy ˃5 years, RP and RT-ADT still yielded similar all-cause death, LRR, and DM, although RP reduced the risk of BF compared with RT-ADT. Given the possible complications and mood adverse effects after salvage treatment [40,41,42,43,44], RP may be the superior choice for relatively young healthy patients with HR/VHR-LPC (Table 3). However, this suggestion should be confirmed by the results of a well-designed RCT. 

Several risk classification tools are available for classifying patients with localized PC and provide data for treatment decisions [5,6,8,47,48,49]. However, the most commonly used risk classification for PC in Taiwan is the NCCN risk classification [8]. In one population-based comparison study, the NCCN risk group system exhibited superior discriminatory ability for predicting PC-specific mortality compared with the EAU risk group system [50]. In our study, we used the NCCN risk group system to classify the patients with LPC. However, reclassification of patients with NCCN HR/VHR-LPC by the EAU risk group system may have the potential to determine the patients with the highest risk of disease progression. If the patients with NCCN HR/VHR-LPC were stratified by the EAU risk group system, 7.5% of the patients in the RP group and 12.6% of the patients in the IMRT plus long-term ADT group would be classified as locally advanced (Table 1). After multivariate Cox proportional hazards regression analysis, EAU-locally advanced PC was a risk factor for disease progression (Table 2, Table 3, Table 4 and Table 5). This finding indicated that if the patients with NCCN HR/VHR-LPC were classified as EAU-locally advanced, these patients would require more aggressive therapy and close follow-up. 

Our results demonstrated that patients treated at academic centers experienced lower all-cause death, BF, and LRR than those treated at nonacademic centers (Table 2, Table 3 and Table 4). This finding is compatible with a relevant study that focused on RP for patients with PC [20]. Additional studies have compared the PSA recurrence rate in patients with PC treated with RP between high-volume and low-volume centers, and these studies have found that fewer PSA recurrences and distant metastases were identified in high-volume centers regardless of whether the treatment was IMRT or RP [21,51]. For patients treated with EBRT plus ADT, the patients who received treatment at a center with a high-volume radiation facility had longer overall survival [52]. The same finding was demonstrated in patients with diffuse large B-cell lymphoma; high-risk patients treated at academic centers had longer overall survival [53]. These data highlight the essential role of facility volume for oncological outcomes in patients with PC. Multivariate Cox proportional hazards regression analysis of all-cause death (Table 2) showed a statistically significant difference (*p* = 0.0026) among patients treated at different hospitals (hospital areas). The hospital areas in Taiwan were associated with rural and urbanized regions in Taiwan. The urbanization grades in Taiwan are North, Central, South, and East Taiwan, in order. In other words, North Taiwan is the most urbanized area in Taiwan. Our results showed that the mortality rate of relatively young men with HR/VHR-LPC and a life expectancy ˃ 5 years were proportion to urbanization grade, whatever the RP and RT-ADT. Our outcomes were also compatible with the previous studies in which the high urbanization grade was associated with low mortality of prostate cancer [54,55]. 

On the risk of radiation-induced second cancer, given that the study presented here compares radiotherapy vs. surgery in relatively young men that have a long-enough lifespan to develop such long-term effects. For relatively young age patients with a longer life expectancy, this aspect might further influence treatment-related decisions among prostate cancer patients with a high risk to develop second malignancies after receiving radiation treatment for the primary tumor [56,57,58]. Moreover, photon exposure (such as IMRT) has a complex radiobiology that influences long-term effects in patients treated for primary cancers. IMRT is likely to almost double the incidence of second malignancies compared with conventional RT [59,60]. The numbers of second malignancies may be larger for longer survival (or for younger patients) using IMRT. Therefore, RP is not only superior to RT-ADT in BF but also results in a low risk of radiation-induced second cancer in these relatively young men with HR/VHR-LPC and a longer life expectancy life.

In comparison to the other National cancer registry-based reports like the Danish Prostate Cancer Registry, Cancer Registry of Norway, and United in Fight against prOstate cancer (UFO) registry [61,62,63], there was more information, consistent treatments, and the same risk stratification as using the NCCN risk stratification, similar ADT duration, sufficient radiation dosage, and the same radiation technique in our TCRD study. Moreover, there was no PSM design in the previous studies, and the most common concern was selection bias among patients from the aforementioned Cancer Registry database [61,62,63] who are receiving RP and RT because of differing backgrounds.

The current manuscript includes totally different populations and outcomes from our previous study [2]. The population, cT stages, PSA, Gleason scores grade, treatments, ADT use, surgical difficulties and complications, life expectancy, and numbers of unfavorable intermediate-risk groups are very different from high- to very high-risk groups. For example, the duration of ADT use for the intermediate-risk group (4–6 months) [2] is different from high- to very high-risk group (1.5 years at least). Moreover, the complications and difficulties of RP were also different between the intermediate groups and high-to very high-risk groups. In addition, the indications of curative treatments for intermediate groups and high- to very-high risk groups were also different. Curative treatments are indicated for a life expectancy with > 10 years and > 5 years for intermediate groups and high- to very high-risk groups, respectively [2]. Therefore, to clarify that the optimal therapeutic treatments of RP or IMRT-ADT are very important between different populations, including intermediate-risk groups and high- to very high-risk groups. Finally, the outcomes were also different in the two studies. RP is only superior to IMRT plus long-term ADT in BF for high- to very high-risk groups in the current study, although RP is superior to IMRT plus short-term ADT in overall survival, BF, LRR, and DM for intermediate-risk groups [2]. In summary, the population, treatments, and outcomes were totally different in the two studies. Therefore, we still think the current study is worthy for valuable clinical references for high- to very-high risk groups.

The strength of our study was that it is the largest and first head-to-head PSM study to compare the detailed oncologic outcomes of RP and high-dose IMRT plus long-term ADT for relatively young patients with NCCN HR/VHR-LPC. Moreover, consistent RT techniques, similar irradiation doses, and homogenous durations of ADT use were employed in this study. Additional potential cofounding factors of BF were well-matched through PSM in our study and indicated balance (Table 1). Our study is the first study to demonstrate a statistical difference in BFFS between RP or high-dose IMRT plus long-term ADT for relatively young patients with NCCN HR/VHR-LPC, but no significant differences were observed regarding all-cause death, LRR, or DM. Our findings may be valuable in shared decision-making between physicians and young patients with NCCN HR/VHR-LPC when selecting between RP or high-dose IMRT plus long-term ADT. In future clinical trials, the oncologic outcomes of RP and high-dose IMRT plus long-term ADT determined herein could be referenced for further risk management in young patients with NCCN HR/VHR PC.

Our study has some limitations. First, brachytherapy was not included in this study because of its lack of favor in Taiwan. The mainstream treatments for localized PC in Taiwan are RP and EBRT. Second, our entire study population was Asian. The results should be cautiously extrapolated to other races. Third, this study did not include possible risk factors regarding all-cause death, such as lifestyle, dietary habits, or body mass index, that might contribute to a high incidence of mortality as a competing risk factor of BF. However, only 5% mortality rate was noted between the two groups and did not reach statistical significance. The potential competing risk of all-cause death for BF could be disregarded. Moreover, BF was estimated using a proportional subdistribution hazard regression model to overcome the competing risk of death in the analysis of time-to-event data [64,65]. Thus, in real-world applications, RP might be associated with BFFS rather than high-dose IMRT with long-term ADT for patients with NCCN HR/VHR-LPC. Finally, this study was a retrospective population cohort study. A prospective RCT is recommended to define the optimal localized treatment for patients with NCCN HR/VHR-LPC. 

## 5. Conclusions

Relatively young patients with NCCN HR/VHR-LPC who received either RP or high-dose IMRT plus long-term ADT had similar oncological outcomes. Additionally, RP demonstrated lower BF.

## Figures and Tables

**Table 1:** Propensity score-matched demographic and clinic characteristics of young patients with high- to very high-risk prostate adenocarcinoma.

		Prostatectomy N = 481		High-Dose IMRT + Long-Term ADT N = 215		
		n	(%)	N	(%)	*p*-Value
Age	Mean (SD)	62.6	(2.3)	62.3	(2.3)	0.3378
	Median (IQR, Q1–Q3)	63	(58–65)	63	(59–65)	
	20–59	73	(15.2)	29	(13.5)	0.9833
	60–65	408	(84.8)	186	(86.5)	
Years of diagnosis	2011–2012	67	(13.9)	30	(14.0)	0.9953
	2013	79	(16.4)	32	(14.9)	
	2014	96	(20.0)	42	(19.5)	
	2015	120	(24.9)	54	(25.1)	
	2016	119	(24.7)	57	(26.5)	
CCI scores	0	220	(45.7)	99	(46.0)	0.9279
	1	124	(25.8)	53	(24.7)	
	2+	137	(28.5)	63	(29.3)	
Myocardial infarction		5	(1.0)	4	(1.9)	0.7702
Congestive heart failure		20	(4.2)	8	(3.7)	0.4891
Peripheral vascular disease		10	(2.1)	5	(2.3)	0.8838
Cerebrovascular disease		26	(5.4)	18	(8.4)	0.3550
Chronic pulmonary disease		60	(12.5)	24	(11.2)	0.5333
Diabetes		132	(27.4)	56	(26.0)	0.5507
Hypertension		244	(50.7)	115	(53.5)	0.7777
Income	Very low	155	(32.2)	68	(31.6)	0.6764
	Low	175	(36.4)	81	(37.7)	
	Middle	98	(20.4)	38	(17.7)	
	High	53	(11.0)	28	(13.0)	
Hospital area	North	259	(53.8)	109	(50.7)	0.9569
	Central	103	(21.4)	45	(20.9)	
	South	110	(22.9)	54	(25.1)	
	East	9	(1.9)	7	(3.3)	
Hospital level	Medical center	289	(60.1)	113	(52.6)	0.5018
	Others	192	(39.9)	102	(47.4)	
cT-stage	cT1	176	(36.6)	75	(34.9)	0.9798
	cT2a	115	(23.9)	52	(24.2)	
	cT2b	24	(5.0)	10	(4.7)	
	cT2c	139	(28.9)	57	(26.5)	
	cT3a	27	(5.6)	21	(9.8)	
Gleason score	≤5	5	(1.0)	6	(2.8)	0.6573
	6	58	(12.1)	54	(25.1)	
	7	289	(60.1)	106	(49.3)	
	8	86	(17.9)	31	(14.4)	
	9+	33	(6.9)	13	(6.0)	
	Missing	10	(2.1)	5	(2.3)	
Grade group	1–2	12	(2.5)	5	(2.3)	0.2556
	3	60	(12.5)	59	(27.4)	
	4	360	(74.8)	134	(62.3)	
	5	49	(10.2)	17	(7.9)	
Preoperative PSA (ng/mL)	0–5	81	(16.8)	31	(14.4)	0.6906
	5–10	148	(30.8)	56	(26.0)	
	10–20	160	(33.3)	65	(30.2)	
	20+	45	(9.4)	43	(20.0)	
	Missing	47	(9.8)	20	(9.3)	
EAU risk group	Localized intermediate	203	(42.2)	77	(35.8)	0.8815
	Localized high	242	(50.3)	111	(51.6)	
	Localized advanced	36	(7.5)	27	(12.6)	
Follow-up time, months	Mean (SD)	60.2	(17.6)	59.9	(17.3)	
All-cause death		27	(5.6)	12	(5.6)	0.5704
Biochemical recurrence		102	(21.2)	84	(39.1)	<0.0001
Locoregional recurrence		27	(5.6)	14	(6.5)	0.9982
Distant metastasis		31	(6.4)	16	(7.4)	1.0000

IQR, interquartile range; SD, standard deviation; RP, radical prostatectomy; T, tumor; cT, clinical tumor stage; PSA, prostate-specific antigen; EAU, European Association of Urology; IMRT, intensity-modulated radiotherapy; ADT, antiandrogen therapy; N, numbers; AJCC, American Joint Committee on Cancer; CCI, Charlson comorbidity index.

**Table 2:** Multivariate Cox proportional hazards regression analysis of all-cause death of young patients with high- to very high-risk prostate adenocarcinoma.

Covariates		Adjusted HR *	(95% CI)	*p*-Value
Curative treatment	Radical prostatectomy	ref		0.5640
	High-dose IMRT + long-term ADT	1.20	(0.65–2.24)	
Age	20–59	ref		0.6834
	60–65	1.18	(0.54–2.58)	
Years of diagnosis	2011–2012	ref		0.4500
	2013	1.30	(0.59–2.87)	
	2014	0.55	(0.21–1.45)	
	2015	0.92	(0.37–2.26)	
	2016	0.82	(0.31–2.18)	
CCI scores	0	ref		0.1043
	1	1.10	(0.51–2.38)	
	2+	2.30	(0.96–5.54)	
Congestive heart failure		1.37	(0.44–4.32)	0.5865
Peripheral vascular disease		0.00	-	0.9797
Cerebrovascular disease		1.10	(0.48–2.53)	0.8199
Chronic pulmonary disease		0.60	(0.22–1.69)	0.3353
Diabetes		1.20	(0.59–2.45)	0.6142
Hypertension		1.37	(0.78–2.40)	0.2762
Income	Very low	ref		0.3395
	Low	1.41	(0.74–2.68)	
	Middle	0.94	(0.43–2.08)	
	High	0.64	(0.24–1.69)	
Hospital level	Academic centers	ref		0.0129
	Nonacademic centers	2.01	(1.16–3.50)	
Hospital area	North	ref		0.0026
	Central	1.64	(0.82–3.28)	
	South	2.20	(1.13–4.31)	
	East	7.68	(2.51–23.55)	
cT-stage	cT1	ref		0.2690
	cT2a	1.02	(0.51–2.02)	
	cT2b	0.67	(0.22–2.02)	
	cT2c	0.47	(0.21–1.07)	
	cT3a	0.38	(0.08–1.74)	
EAU risk group	Localized intermediate	ref		0.0454
	Localized high	1.57	(0.81–3.06)	
	Localized advanced	2.55	(1.36–5.18)	

RP, radical prostatectomy; T, tumor; cT, clinical tumor stages; PSA, prostate-specific antigen; EAU, European Association of Urology; IMRT, intensity-modulated radiotherapy; ADT, antiandrogen therapy; AJCC, American Joint Committee on Cancer; CCI, Charlson comorbidity index; CI, confidence interval; aHR, adjusted hazard ratio; Ref, reference group; NTD, New Taiwan Dollars. * All covariates in Table 2 were adjusted.

**Table 3:** Multivariate Cox proportional hazards regression analysis of biochemical recurrence in young patients with high- to very high-risk of prostate adenocarcinoma.

Covariates		Adjusted HR *	(95% CI)	*p*-Value
Curative treatment	Radical prostatectomy	ref		<0.0001
	IMRT + long-term ADT	2.03	(1.56–2.65)	
Age	20–59	ref		0.6054
	60–69	1.08	(0.82–1.42)	
	70–80			
	80+			
Years of diagnosis	2011–2012	ref		0.3193
	2013	0.81	(0.56–1.16)	
	2014	0.89	(0.62–1.27)	
	2015	0.85	(0.59–1.22)	
	2016	0.67	(0.45–0.98)	
CCI scores	0	ref		0.6576
	1	1.12	(0.83–1.52)	
	2+	1.20	(0.79–1.83)	
Congestive heart failure		0.74	(0.38–1.44)	0.3760
Peripheral vascular disease		0.51	(0.16–1.64)	0.2569
Cerebrovascular disease		1.07	(0.67–1.70)	0.7711
Chronic pulmonary disease		0.92	(0.58–1.45)	0.7106
Diabetes		0.97	(0.68–1.37)	0.8568
Hypertension		0.93	(0.73–1.17)	0.5365
Income	Low	ref		0.6583
	Very Low	1.06	(0.78–1.43)	
	Middle	1.13	(0.82–1.55)	
	High	0.91	(0.66–1.26)	
Hospital level	Academic centers	ref		0.0073
	Nonacademic centers	1.37	(1.09–1.73)	
Hospital area	North	ref		0.1560
	Central	1.05	(0.93–1.79)	
	South	1.11	(0.83–1.49)	
	East	1.50	(0.70–3.18)	
cT-stage	cT1	ref		0.4036
	cT2a	1.04	(0.78–1.39)	
	cT2b	1.08	(0.71–1.64)	
	cT2c	1.18	(0.41–1.81)	
	cT3a	1.26	(0.24–1.99)	
EAU risk group	Localized intermediate	ref		<0.0001
	localized-high	2.18	(1.63–2.91)	
	Localized advanced	3.41	(1.59–7.32)	

RP, radical prostatectomy; T, tumor; cT, clinical tumor stage; PSA, prostate-specific antigen; EAU, European Association of Urology; IMRT, intensity-modulated radiotherapy; ADT, antiandrogen therapy; AJCC, American Joint Committee on Cancer; CCI, Charlson comorbidity index; CI, confidence interval; aHR, adjusted hazard ratio; Ref, reference group; NTD, New Taiwan Dollars. * All covariates mentioned in Table 2 were adjusted.

**Table 4:** Multivariate Cox proportional hazards regression analysis of locoregional recurrence in young patients with high- to very high-risk prostate adenocarcinoma.

Covariates		Adjusted HR *	(95% CI)	*p*-Value
Curative treatment	Radical prostatectomy	ref		0.3524
	IMRT + long-term ADT	0.88	(0.67–1.06)	
Age	20–59	ref		0.5068
	60–65	0.87	(0.57–1.32)	
Years of diagnosis	2011–2012	ref		0.6379
	2013	1.57	(0.81–3.03)	
	2014	1.26	(0.66–2.41)	
	2015	1.38	(0.72–2.63)	
	2016	1.08	(0.53–2.20)	
CCI scores	0	ref		0.1806
	1	0.58	(0.33–1.04)	
	2+	0.66	(0.32–1.36)	
Congestive heart failure		2.10	(0.73–6.01)	0.1665
Peripheral vascular disease		0.90	(0.67–1.31)	0.4021
Cerebrovascular disease		1.61	(0.70–3.71)	0.2584
Chronic pulmonary disease		0.90	(0.37–2.20)	0.8124
Diabetes		1.32	(0.75–2.33)	0.3324
Hypertension		0.80	(0.54–1.20)	0.2893
Income	Very Low	ref		0.1690
	Low	0.74	(0.44–1.27)	
	Middle	1.05	(0.63–1.72)	
	High	0.61	(0.36–1.04)	
Hospital level	Academic centers	ref		0.0456
	Nonacademic centers	1.05	(1.00–1.42)	
Hospital area	North	ref		0.9213
	Central	0.90	(0.58–1.40)	
	South	1.04	(0.62–1.76)	
	East	0.62	(0.08–4.54)	
cT-stage	cT1	ref		0.2812
	cT2a	1.00	(0.63–1.60)	
	cT2b	1.03	(0.43–1.60)	
	cT2c	1.06	(0.40–1.63)	
	cT3a	1.11	(0.51–1.78)	
EAU risk group	Localized intermediate	ref		0.0077
	Localized high	1.68	(1.08–2.62)	
	Localized advanced	5.32	(1.44–19.72)	

RP, radical prostatectomy; T, tumor; cT, clinical tumor stage; PSA, prostate-specific antigen; EAU, European Association of Urology; IMRT, intensity-modulated radiotherapy; ADT, antiandrogen therapy; AJCC, American Joint Committee on Cancer; CCI, Charlson comorbidity index; CI, confidence interval; aHR, adjusted hazard ratio; Ref, reference group; NTD, New Taiwan Dollars. * All covariates mentioned in Table 2 were adjusted.

**Table 5:** Multivariate Cox proportional hazards regression analysis of distant metastasis in patients with high- to very high-risk of prostate adenocarcinoma.

Covariates		Adjusted HR *	(95% CI)	*p*-Value
Curative treatment	Radical prostatectomy	ref		0.9176
	IMRT + long-term ADT	1.03	(0.56–1.90)	
Age	20–59	ref		0.7536
	60–69	1.10	(0.62–1.95)	
	70–80			
	80+			
Years of diagnosis	2011–2012	ref		0.2664
	2013	1.66	(0.83–3.31)	
	2014	0.91	(0.42–1.95)	
	2015	0.76	(0.35–1.67)	
	2016	0.97	(0.44–2.16)	
CCI scores	0	ref		0.4698
	1	1.22	(0.63–2.36)	
	2+	1.70	(0.72–4.02)	
Congestive heart failure		0.57	(0.14–2.40)	0.4450
Peripheral vascular disease		1.45	(0.39–5.42)	0.5775
Cerebrovascular disease		1.21	(0.56–2.60)	0.6237
Chronic pulmonary disease		0.98	(0.43–2.25)	0.9626
Diabetes		1.64	(0.85–3.19)	0.1424
Hypertension		1.16	(0.73–1.84)	0.5247
Income	Very Low	ref		0.8722
	Low	1.29	(0.69–2.41)	
	Middle	1.23	(0.63–2.40)	
	High	1.20	(0.60–2.41)	
Hospital level	Medical center	ref		0.1107
	Others	1.49	(0.91–2.43)	
Hospital area	North	ref		0.2710
	Central	1.59	(0.92–2.75)	
	South	2.07	(0.88–3.62)	
	East	3.85	(0.81–4.71)	
cT-stage	cT1	ref		0.4248
	cT2a	1.00	(0.55–1.67)	
	cT2b	1.03	(0.51–1.12)	
	cT2c	1.08	(0.69–1.61)	
	cT3a	1.09	(0.67–2.82)	
EAU risk group	Localized intermediate	ref		0.0114
	localized high	1.26	(1.08–3.17)	
	Localized advanced	3.43	(1.58–4.43)	

RP, radical prostatectomy; T, tumor; cT, clinical tumor stage; PSA, prostate-specific antigen; EAU, European Association of Urology; IMRT, intensity-modulated radiotherapy; ADT, antiandrogen therapy; AJCC, American Joint Committee on Cancer; CCI, Charlson comorbidity index; CI, confidence interval; aHR, adjusted hazard ratio; Ref, reference group; NTD, New Taiwan Dollars. * All covariates mentioned in Table 2 were adjusted.

## Data Availability

Restrictions apply to the availability of these data. Data was obtained from Taiwan Ministry of Health and Welfare and are available from Szu-Yuan Wu with the permission of Institutional Review Board of Tzu-Chi Medical Foundation (IRB109-015-B).

## Abbreviations

RP, radical prostatectomy; PC, prostate cancer; BFFS, biochemical failure-free survival; CI, confidence interval; T, tumor; PSA, prostate-specific antigen; AJCC, American Joint Committee on Cancer; SD, standard deviation; HR, hazard ratio; aHR, adjusted hazard ratio; RCT, randomized controlled trial; NCCN, National Comprehensive Cancer Network; RT-ADT, radiation plus antiandrogen therapy; HR/VHR-LPC, high- or very high-risk localized prostate cancer; HRLPC, high-risk localized prostate cancer; TCRD, Taiwan Cancer Registry database; IMRT, intensity-modulated radiotherapy; LPC, localized prostate cancer; EAU, European Association of Urology; AUA, American Urological Association; EBRT, external beam radiotherapy; ADT, antiandrogen therapy; PSM, propensity score matching; NHIRD, National Health Insurance Research Database; CCI, Charlson comorbidity index; *ICD-10-CM*, *International Classification of Diseases, Ninth Revision, Clinical Modification*; OS, overall survival.
